# Supplementary material for: Investigation of Prognostic Value of Claudin-5, PSMA, and Ki67 Expression in Canine Splenic Hemangiosarcoma
Source: Animals (Basel). 2021 Aug 14;11(8):2406. doi: 10.3390/ani11082406 (PMC8388721; doi:10.3390/ani11082406)
Supplement: Supplementary file 1 [file animals-11-02406-s001.zip › animals-1290933-supplementary.pdf]

Supplementary Table S1. Identification and sequence of the primers used for qPCR.

Expression Product	Gene ID	Expression primer sequence (5`- 3`)
**CLAUDIN-5**	100684266	Forward: CCTTTGTGTTTCTGCTCTCTGA<br>Reverse: CCCAGTGTTCTCCAGTGACTCA
**PSMA**	476775	Forward:TCGATTGCACCCCACTGAT<br>Reverse: GGGCTTTGGAGCTCCTTTGT
**ACTB**	487218	Forward: TACAGCTTCACCACCACTGC<br>Reverse: CTTCTCCATGTCGTCCCAGT
**GAHPD**	403755	Forward: CATCAACGGGAAGTCCATCT<br>Reverse: TACTCACCACCAGCATCACC
**HPRT**	442945	Forward: CGGCTTGCTCGAGATGTGAT<br>Reverse: GCACACAGAGGGCTACGATGT
**RPL8**	475130	Forward: CCATGAATCCTGTGGAGC<br>Reverse: GTAGAGGGTTTGCCGATG
**RPS19**	476450	Forward: CCTTCCTCAAAAAGTCTGGG<br>Reverse: GTTCTCATCGTAGGGAGCAAG
**RPS 5**	476366	Forward: TCACTGGTGAGAACCCCCT<br>Reverse: CCTGATTCACACGGCGTAG

Supplementary Table S2. Specification of the immunohistochemistry protocol used in this research**.**

Antibody	Clone	Peroxidase Blockage	Concentration	DAB
**Ki-67**	Clone MIB-1(Dako Ominis, Santa Clara, CA)	1 hour	1: 50	5min
**CLAUDIN-5**	Monoclonal mouse anti-human antibodies (Zymed Inc, San Francisco, CA, USA)	1 hour	1:200	1min
**PSMA**	Anti-human mouse (Novus Biological, Littleton, CO)	30 min	1:2000	1min

PSMA= Prostatic specific membrane antigen.
